# In vitro Anti-cholinesterase and Anti-oxidant Activity of Three Standardised Polyherbal Products Used for Memory Enhancing in Ethnomedicine of South-East Nigeria

**DOI:** 10.21315/mjms2018.25.2.4

**Published:** 2018-04-27

**Authors:** Lucky Legbosi Nwidu, Ekramy Elmorsy, Wayne Grant Carter

**Affiliations:** 1 Department of Experimental Pharmacology and Toxicology, Faculty of Pharmaceutical Sciences, University of Port Harcourt, Port Harcourt, Nigeria; 2 Department of Forensic Medicine and Clinical Toxicology, Faculty of Medicine, Mansoura University, Egypt; 3 School of Medicine, University of Nottingham, Royal Derby Hospital Centre, Derby, UK

**Keywords:** anti-cholinesterases, anti-oxidants, memory improvement, phenolics, polyherbal

## Abstract

**Background:**

Polyherbal standardised extracts used in ethnomedicine of Eastern Nigeria for memory improvements were evaluated for anti-cholinesterases and anti-oxidant properties.

**Methods:**

Anti-cholinesterase, anti-oxidant, and total phenolic and flavonoid contents were established using standard procedures.

**Results:**

The three polyherbal extracts exhibited significant concentration dependent acetylcholinesterase (AChE) inhibitory activity (*P* = 0.001). The highest AChE inhibition was observed with the Neocare Herbal Tea (NHT) with 99.7% (IC50 = 324 μg/mL); whereas the Herbalin Complex Tea (HCT) and Phytoblis Herbal Tea (PHT) exhibited 73.8% (IC50 = 0.2 μg/mL) and 60.6% (IC50 = 0.7 μg/mL) inhibition, respectively, relative to eserine at 100% inhibition (IC50 = 0.9 μg/mL) at 200 μg/mL. The order of percentage increase in inhibition of AChE was NHT > HCT > PHT; while the order of decrease in potency was HCT > PHT > NHT.

Radical scavenging activities of HCT, NHT and PHT were 82.13% (IC50 = 0.08 μg/mL), 77.43% (IC50 = 0.01 μg/mL) and 76.28% (IC50 = 0.3 μg/mL), respectively, at 1 mg/mL concentrations. The reducing power revealed a dose-dependent effect, with NHT > PHT > HCT. The order of total phenolics content in the extracts were PHT > HCT > NHT, and for total flavonoids content: PHT > NHT > HCT.

**Conclusion:**

The three polyherbal standardised products possess significant acetylcholinesterase inhibitory activity and secondary metabolites that could collectively contribute to their memory-enhancing effects.

## Graphical abstract

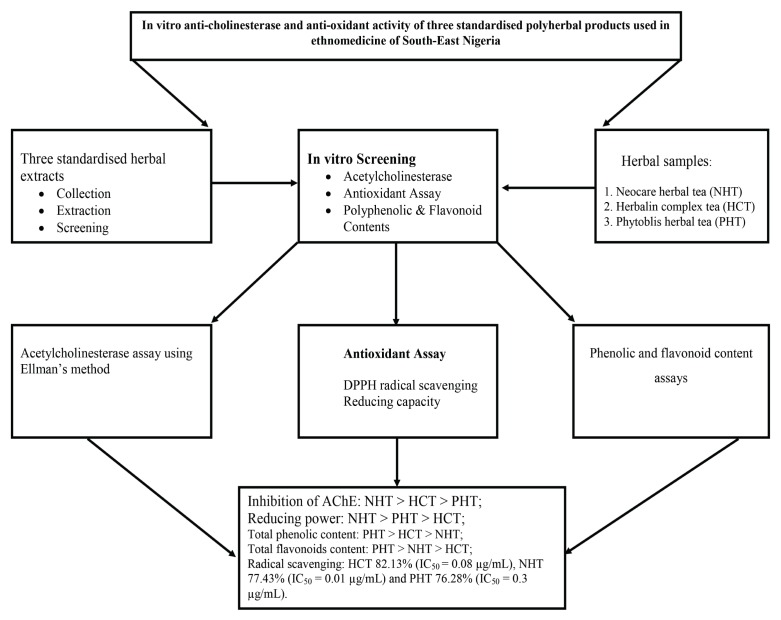

## Introduction

Herbal medicines have witnessed a substantive growth in popularity underscored by their efficacy in alleviating a plethora of ailments. This increased usage likely reflects their availability, acceptability, and limited side effects (1). Basic healthcare needs, such as drugs, are in short supply in many developing countries and a large proportion of the population patronise traditional and alternative medicine practitioners. Attractive packaging and unregulated advertisement of herbal products, most especially their presentation as supplements or herbal teas have given herbal products worldwide appeal. Synthetic molecules have yet to completely attenuate medical conditions including dementias, such as Alzheimer disease (AD), and Parkinson disease (PD), hence the need for the continuous screening of indigenous medicines to discover suitable drugs to alleviate disease symptoms.

The pathophysiology of AD is complex and insidious due to multifaceted mechanisms. In AD, acetylcholine (ACh) deficiency underlies memory and learning impairment, and this has led to the ‘cholinergic hypothesis’ of AD. ACh is stored in vesicles and released at nerve terminals after nerve cell depolarisation. ACh crosses the synaptic cleft where it binds to post-synaptic ACh receptors. The levels of synaptic ACh are regulated by acetylcholinesterase (AChE), hence this has led to the development of several classes of AChE inhibitors, such as donepezil, galantamine, and rivastigmine, as treatments to sustain ACh signaling and limit the cholinergic deficit in mild to moderate AD (2–8). The use of these drugs and memantine, a non-competitive N-methyl-D-aspartate receptor antagonist, may limit disease severity, but the development of new drugs for AD has been limited.

Another defining postulate of AD pathogenesis and progression is the ‘amyloid-beta hypothesis’ which attributes the probable cause of AD to the distorted production, aggregation, and deposition of beta-amyloid peptide (Aβ) into neuritic plaques (8). In addition, the ‘tau hypothesis’ suggests that the formation of intracellular neurofibrillary tangles that are composed of hyper-phosphorylated tau protein is also neurotoxic and contributory to AD pathogenesis and/or progression (9, 10). Consideration of these paradigms has highlighted the quest for disease-modifying drugs with broad activities, but few have preceded beyond phase 3 clinical trials due to a lack of efficacy and patient neurotoxicity (11).

AD neurotoxicity may also be exacerbated via the generation of reactive oxygen species (ROS), as a consequence of both an accelerated generation of ROS and a gradual decline in cellular anti-oxidant defense mechanisms with senility. Hence mitochondria-targeted anti-oxidants have proven to be successful in counteracting Aβ toxicity in animal models, and in improving cognitive function and behavioral deficits in patients with mild to moderate AD (12, 13). Therefore, AD pathogenesis may involve potential interactions between Aβ, oxidative stress, and neurofibrillary tangles that collectively contribute to disease progression and complexity (8, 14, 15, 16). Other related emerging biochemical and metabolic pathways and targeted drug foci are also emerging (17, 18).

Today, most reports indicate that the etiology of AD, and many other neurodegenerative disorders, is based on multiple causal factors rather than a single etiopathological mechanism (19, 20). Underpinning the multifactorial nature of AD, a polypharmacy-based therapy with multipronged targets (21) might overcome some of the major limitations of currently available drugs, whose innovation has traditionally relied upon the one-molecule, one-target paradigm (22). Hence the use of drug entities that display multiple target specificity may be of improved therapeutic benefit than single-target drugs or combination therapy (22, 23). Indeed, recent drug trials have suggested that AD treatment using a poly-pharmacy product may better address the multiple etiopathology of AD (24).

Molecules with anti-cholinesterases and anti-oxidant capacity could likely provide suitable candidate drugs to modify AD pathology. However, direct synthetic anti-oxidants usage may be excluded due to potential adverse effects (25). Plant based medicines used as natural products are reported to possess beneficial synergistic activities when used as combinations (26). Polyherbal drugs have been widely used in adjuvant therapy in the management of chronic diseases (27, 28). Therefore, it becomes imperative to screen polyherbal products to ascertain claims of efficacy, safety and verify side effect profiles. Medicinal plant components are rich sources of secondary metabolites that act synergistically at multiple targets to provide important therapeutic effects.

Hence, three polyherbal formulations: Neocare Herbal Tea (NHT), Herbalin Complex Tea (HCT), and Phytoblis Herbal Tea (PHT) that have been suggested to combat memory impairment were screened for possible anti-cholinesterase, and anti-oxidant effects. The rationale for selection of these three polyherbal products also relies on the documented multiple pharmacological activities of the individual components of each of these herbal formulations. This is the first scientific report on the activities of these standardised polyherbal extracts in vitro.

## Materials and Methods

### Chemicals and Reagents

Acetylthiocholine iodide (ATCI), L-ascorbic acid, bovine serum albumin (BSA), dimethyl sulphoxide (DMSO), 2,2-diphenyl-1-picrylhydrazyl (DPPH), 5,5,-dithiobis[2-nitrobenzoic acid] (DTNB), eserine, ferric chloride, Folin-Ciocalteau reagent, gallic acid, potassium ferricyanide, β-tocopherol, and trichloroacetic acid were all purchased from Sigma, UK. All other chemical and reagents used in this study were of analytical grade and were obtained from Sigma, UK unless stated otherwise.

### Plants Herbal Samples and Extraction

A standardised herbal extract is an herb extract that has one or more components present in a specific guaranteed amount usually expressed as a percentage. The polyherbal standardised extracts used in this investigation were herbal extracts having two or more different plant materials in a specific guaranteed amount, expressed as percentages. The purpose behind the polyherbal standardisation is to assure to the consumer that the product retains the same chemical make-up and that this is consistent from batch to batch. This practice has developed out of the drug model in which reproducible amounts of different herbs are consistently used for every product dispensed. This differs from pharmacy practices in which scientists identify the components of a plant(s) that have definite pharmacological activity in the body and formulate the bioactive agent(s).

The standardised polyherbal extracts were obtained from a herbalist during a herbal exposition at the Herbfest conference held in October in Abuja, Nigeria in 2014. The crude powdered extract of NHT and PHT (100 g each) was immersed in 500 mL of methanol for 72 h. For the HCT, 80 g was immersed in 400 mL of methanol. In each case the mixture was shaken twice daily before filtration through a double layer gauze to obtain a supernatant that was then evaporated to dryness at 50 °C in vacuo. The dried methanolic extract yielded 4.22%, 5.53% and 5.24% of NHT, HCT and PHT, respectively (Table 3.1). The extracts were stored at 4 °C until required. Each extract was reconstituted with double distilled water before it was used in experimental assays. The phytocomposition per pack of each polyherbal formula is shown in Table 1.

### Determination of Acetylcholinesterase Inhibitory Activity Using a Microtiter Plate Assay

Acetylcholinesterase (AChE) activity was measured in a 96-well microtiter plate assay based on the method of Ellman et al. (29), as published previously (30). For each assay data point, 50 μL of 3 mM 5,5′-dithiobis-(2-nitrobenzoic acid) (DTNB), 50 μL of AChE (1 mg/mL) (Sigma, C3389) or rat brain homogenate (prepared according to previous publications (31, 32)), 35 μL of 50 mM Tris/ HCl pH 8.0, and 40 μL of polyherbal extract were mixed and incubated at 37 °C. The assay was initiated by addition of 25 μL of 15 mM acetylthiocholineiodide (ATCI), with production of 5-thio-2-nitrobenzoate anion read at 412 nm every 30 sec for 10 min using a Spectramax microplate reader (ThermoFisher, UK). Assay reactions with polyherbal extracts were all performed in triplicate at concentrations of 200 μg/mL, 20 μg/mL, 2 μg/mL, 0.2 μg/mL and 0.02 μg/mL. A negative control assay performed in the absence of AChE provided a reagent blank. Eserine (Sigma, E8375) was used as a positive control to inhibit electric eel or rat brain AChE in a dose-dependent manner. The percentage inhibition of AChE by polyherbal extract was calculated relative to inhibition by eserine, with the herbal extract concentration producing 50% inhibition (IC_50_) of AChE calculated using GraphPad Prism (version 5.03, Inc., 2010, San Diego California USA) via non-linear regression analysis.

### Assessment of 2,2-Diphenyl-1-Picrylhydrazyl Radical Scavenging Activity

A 2,2-diphenyl-1-picrylhydrazyl (DPPH) assay was employed, to determine by a spectroscopic method, the anti-oxidant capability via free radical scavenging activity of polyherbal extracts using a method described previously (33). Stock solutions of each of the polyherbals (5 mg/mL) were diluted to final concentrations of 200 μg/mL, 100 μg/mL, 50 μg/mL, 25 μg/ mL, 12.5 μg/mL, and 6.25 μg/mL in ethanol. One hundred and sixty microlitres of 0.1 mM DPPH in ethanol was added to 20 μL of extract or fraction, or Vitamin E (as a positive control), and the material was mixed with 20 μL of water. β-tocopherol was used as control and assayed over a concentration range of 1.56 mg/mL, 0.78 mg/mL, 0.39 mg/mL, 0.195 mg/mL and 0.0975 mg/mL. The mixture was incubated at 37 °C for 40 min in the dark, before reading the absorbance at 517 nm using a Spectramax microplate reader. Experimental blanks were performed in the absence of polyherbal extracts. The percentage anti-oxidant activity was estimated as the percent DPPH radical scavenging activity. All tests were performed in triplicate and inhibition percentages reported as means (± SD).

### Assessment of Reducing Power Capacity

The reducing capacity of polyherbal methanolic extracts was evaluated via their ability to reduce ferric iron (Fe^3+^) to ferrous iron (Fe^2+^). Concentrations of polyherbal extracts were prepared across a concentration range of 6.25 μg/mL–50 μg/mL. Aliquots of 4 μL of 5 mg/mL of each plant sample were added to 400 μL of phosphate buffer pH 7.4 and 250 μL of 1% potassium ferricyanide and incubated at 50 °C for 20 min. Then 250 μL of 10% trichloroacetic acid was added, and after vortexing, the sample was centrifuged at 3000 rpm for 10 min. One hundred microlitres of the supernatant was removed and mixed with a similar volume of water and added to a microtiter plate. Twenty microlitres of freshly prepared ferric chloride solution was added, resulting in the formation of Perls’ Prussian blue, with absorbance read at 700 nm using a Spectramax plate reader (30, 33). A blank was prepared without addition of anti-oxidant. All assay points were conducted in triplicates. L-ascorbic acid was used as a positive control anti-oxidant compound. The percentage increase of reduction activity with increasing concentration of the extracts was assessed.

### Determination of Total Phenolic and Total Flavonoid Content

Quantitation of total phenolics in polyherbal extracts utilised the Folin-Ciocalteau reagent (FCR) method as detailed in previous publications (30, 33). Twenty microlitres of polyherbal extracts with concentration range of 1 μg/mL–100 μg/mL, was mixed with 90 μL of double distilled water, quickly followed by the addition of 30 μL of FCR and the mixture shaken vigorously on a plate reader. Within 10 min, 60 μL of 10% Na2CO3 solution was added and the material incubated at 40 °C in a shaking incubator. After 40 min the absorbance of the mixture was read in a Spectramax plate reader at 760 nm. Gallic acid (Sigma, CAS14991-7) was similarly processed over a concentration range of 0.1 mg/mL–0.5 mg/mL as a positive control, and was used to generate a calibration curve for quantification of total phenolic content in polyherbal extracts across the concentration range of 0.01 mg/mL–0.05 mg/mL. Total phenolic content was expressed as mg gallic acid equivalents/gram of plant extract (mg GAE/g).

Total flavonoid content of polyherbal extracts was determined similar to the method described by Zhishen et al. (34), as previously published (30), using quercetin as a reference compound. Twenty microlitres of polyherbal extract (5 mg/mL) in ethanol was mixed in a microplate well with 200 μL of 10% aluminum chloride solution and 1M potassium acetate solution. The mixture was incubated for 30 min at room temperature before measuring the absorbance at 415 nm using a Spectramax plate reader. The total flavonoids content of polyherbal extracts was quantified as mg quercetin equivalent per gram of polyherbal extract (mg QUER equivalent/g).

### Cytotoxicity Assays

#### Cell culture

Hepatocellular carcinoma (HepG2) cells were cultured in Eagle’s minimal essential media (EMEM) with 2 mM Glutamine, 1% Non-Essential Amino Acids (NEAA), 10% Foetal Bovine Serum (FBS), and antibiotics (100 μg/mL penicillin and 100 μg/mL streptomycin). Cells were grown in a humidified atmosphere at 37 °C and 5% CO_2_.

#### Cytotoxicity assays

Cells were seeded at 3 × 10^4^ cells per well in 96-well plates. At ~80%–90% confluence, polyherbal standardised extracts were added to wells at 1 μg/mL, 10 μg/mL, 100 μg/mL, and 1000 μg/mL. After 48 h, treatment media was removed, and cells incubated with fresh media containing 0.5% thiazolyl blue tetrazolium bromide (MTT) reagent (Sigma, M2128). After 4 h at 37 °C in the humidified incubator, the media was replaced with 100 μL of isopropanol and DMSO solution (1:1 (v/v)) and the absorbance read at 540 nm in a Spectramax plate reader. Each assay point was conducted in triplicate. The percentage of cells that were metabolically active was calculated for each concentration of polyherbal methanolic extract.

### Statistical Analysis

Results are expressed as means (± SD). Normality was assessed by the Kolmogorov-Smirnov (K-S) test which was conducted in SPSS. The concentration of agent producing 50% inhibition (IC_50_) was calculated using non-linear regression analysis with the GraphPad Prism (Version 5.03) for Windows (GraphPad Software, Inc., San Diego, CA, USA, www.graphpad.com).

## Results

### Composition of Standardised Herbal Products and Yield of Methanolic Extracts

Polyherbal standardised products: Neocare herbal tea (NHT), Herbalin complex tea (HCT) and Phytoblis herbal tea (PHT) used for memory improvement in ethnomedicine of Eastern Nigeria have three or four herbs. Neocare herbal tea (NHT) contains standardised plants materials such as *Alchornea cordifolia* (Euphurbiaceae) leaves, *Azadirachta indica* (Meliaceae) and *Pteridium aquilinum* (Dennstaedtiaceae); HCT contains *Hippocratea volubilis* (Hippocrateaceae), *Viscum album* (Santalaceae) and *Thymus pulegiodes* (Lamiaceae); PHT contain *Urtica dioica* (Urticaceae) stinging nettles, *Hippocratea volubilis* (Hippocrateaceae), *Thymus vulgaris* (Lamiaceae), and *Aloe vera* (Asphodelaceae). The composition and yield of each extract of these polyherbal products is presented in Table 1.

### Polyherbal Extracts Exhibited Acetylcholinesterase Inhibitory Activity

**Figure 1:**
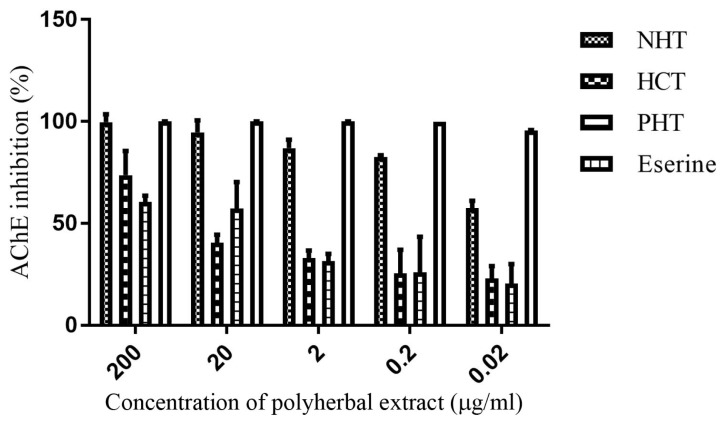
Acetylcholinesterase inhibitory activity of polyherbal extracts. Values presented as mean percentage inhibition ± SD, *n* = 3

The three polyherbal methanolic extracts exhibited significant (*P* = 0.001) concentration dependent AChE inhibitory activity (Figure 1). The polyherbal NHT extract had the highest AChE inhibition of 99.7% (IC_50_ = 324 μg/ mL); HCT demonstrated 73.8% (IC_50_ = 0.2 μg/ mL) and PHT gave 60.6% (IC_50_ = 0.7 μg/mL) inhibition while the pure drug, eserine, 100% inhibition (IC_50_ = 0.6 μg/mL) at 200 μg/mL. The order of percentage increase in inhibition of AChE was NHT > HCT > PHT; while the order of potency decreased according to HCT > PHT > NHT. The IC_50_ values are included in Table 2.

### Polyherbal Extracts Exhibited DPPH Radical Scavenging Activity

**Figure 2:**
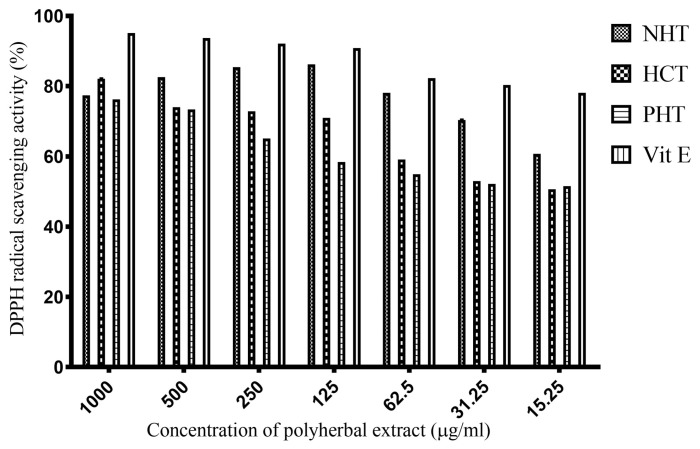
Antioxidant activity of polyherbal extracts. Values presented as mean percentage inhibition ± SD, *n* = 3

The polyherbal methanolic extracts exhibited DPPH radical scavenging activity in a concentration-dependent manner (Figure 2). The percentage DPPH radical scavenging by each of the polyherbal extracts were HCT: 82.13% (IC_50_ = 0.08 μg/mL), NHT: 77.43% (IC_50_ = 0.01 μg/mL) and PHT: 76.28% (IC_50_ = 0.3 μg/mL) inhibition, respectively, at 1 mg/mL concentration. IC_50_ values have been included in Table 2.

### Polyherbal Methanolic Extracts Displayed Reducing (Anti-Oxidant) Activity

**Figure 3:**
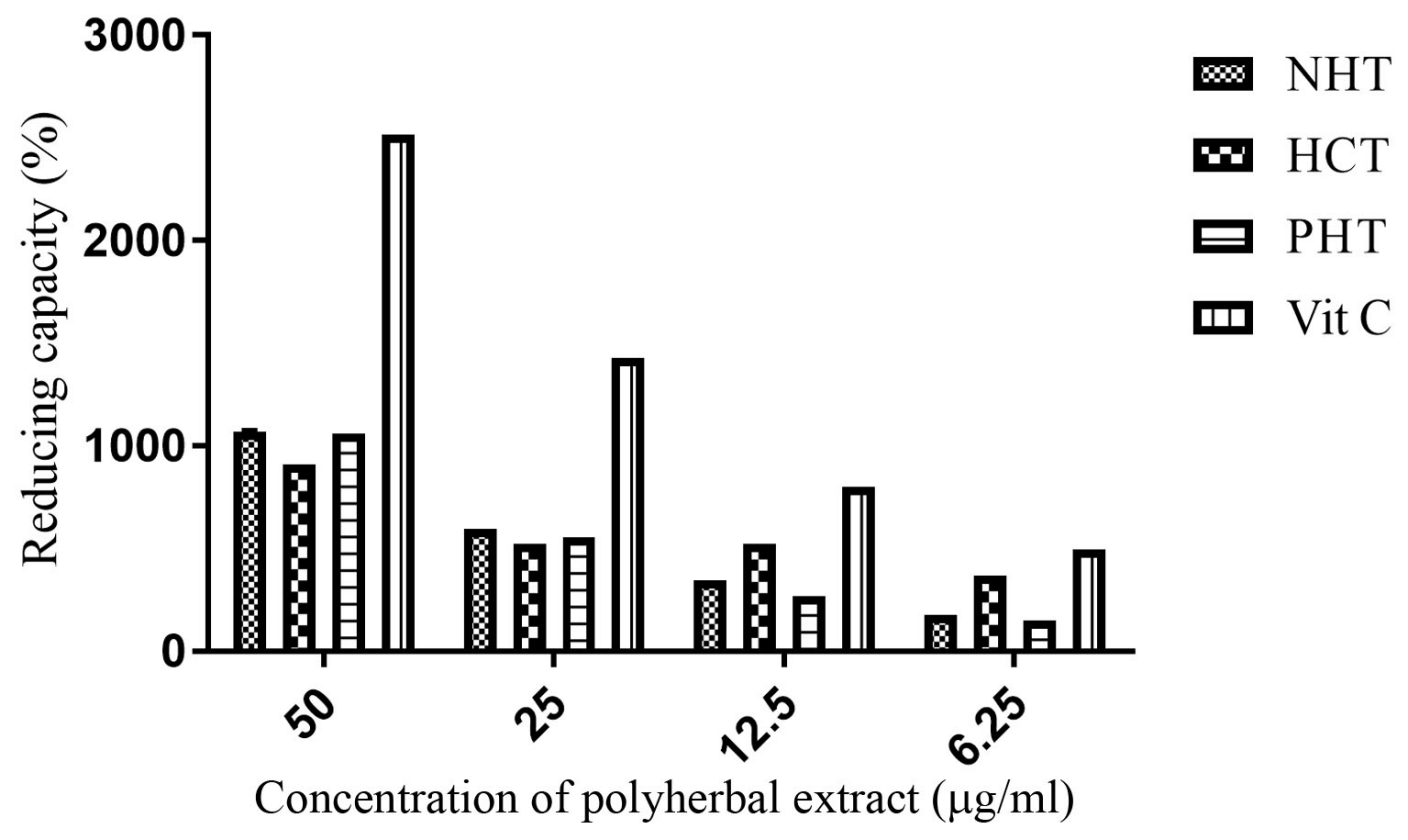
Reducing capacity of polyherbal extracts. Values presented as mean percentage inhibition ± SD, *n* = 3

The polyherbal methanolic extracts demonstrated a concentration-dependent increase of anti-oxidant (reducing) capacity with increasing extract concentration (Figure 3). Reducing power of the different herbal extracts was not as effective as the standard drug, ascorbic acid. The reducing power of the polyherbal extracts was in the order: NHT > PHT > HCT at 50 μg/mL.

### Total Phenolic Content and Total Flavonoid Content of Polyherbal Extracts

**Figure 4:**
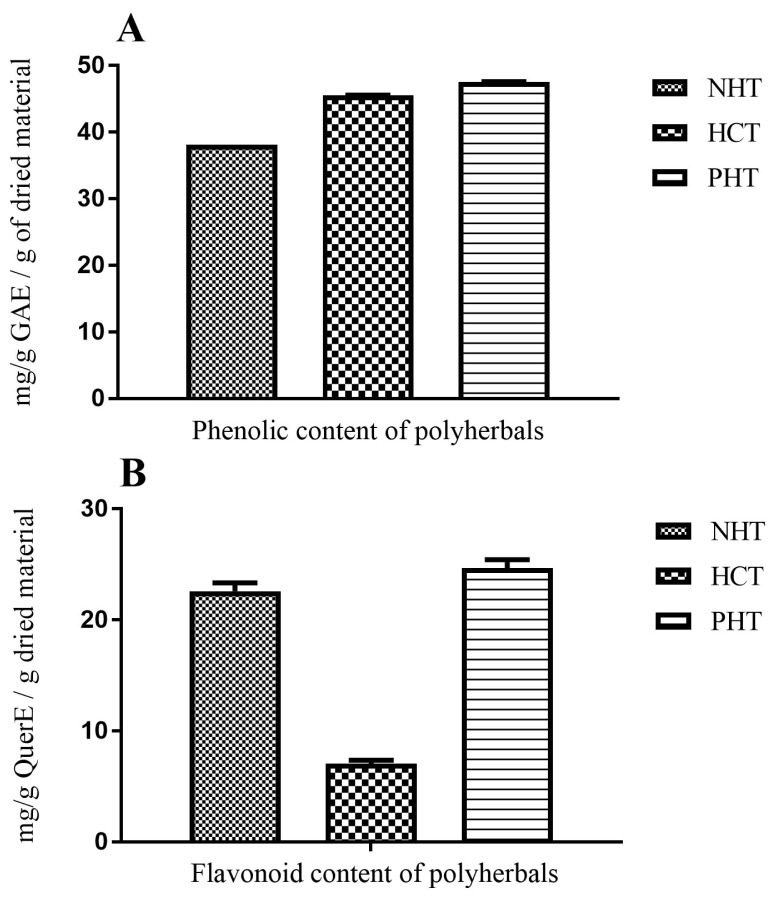
Total phenolic content (A), and total flavonoid content (B) of polyherbal extracts. Values presented as mean ± SD, *n* = 3

The total phenolic content (TPC) of the polyherbal extracts are shown in Figure 4A. The TPC of the extracts were calculated using the standard curve for Gallic acid (y = 0.006x + 0.147; R^2^ = 0.996). The order of TPC in the polyherbal extracts revealed that PHT > HCT > NHT. The results of aluminum chloride colorimetric determination of total flavonoid contents (TFC) are shown in Figure 4B. TFC values were estimated using the standard curve for quercetin (y = 0.0244x + 0.161; R^2^ = 0.974). The results showed that the TFC content was in the order PHT > NHT > HCT and was dose dependent (*P* < 0.0001).

### Polyherbal Extracts are Tolerated by Liver Cells at High Doses

Polyherbal standardised extracts were evaluated for cytotoxicity to human liver HepG2 cells. Cytotoxicity was observed to be concentration-dependent but with significant loss of metabolic activity only at the highest dose employed for all extracts (1000 μg/mL). HepG2 cell metabolic activity at 1000 μg/mL was ~43%, 66%, and 71% for NHT, HCT and PHT, respectively. Cytotoxicity effects of the polyherbal extracts were minimal at lower doses. The IC_50_ viability values for the polyherbal extract were in the order: PHT > HCT > NHT. The cytotoxicity profile of these three standardised herbal extracts was negligible and approximately equipotent at 10 μg/mL. Some extracts, however, moderately increased the metabolic activity of the cells at certain concentrations (Table 3).

## Discussion

Neurodegenerative diseases may be exacerbated by the generation of free radicals and associated cellular redox stress, a process that could be effectively and efficiently ameliorated by herbal products that possess anti-oxidant activity. Ethnomedicine has utilised polyherbal formulations purported to have multiple health benefits (28). Indeed, standardised extracts may have wide acceptance since they can produce reproducible therapeutic effects comparable to crude extracts (28). Clinical effectiveness requires delivery of an active dosage and the use of standardised extracts is an innovative way to assure the delivery of an effective dosage (35).

Several reports have evaluated the phytocomponents of PHT, HCT and NHT, however, prior to this study no data exists on the anti-cholinesterase activity or anti-oxidant effects of these polyherbal combinations. The effect of polyherbal extracts on one of the primary defects in AD; that of a deficit in acetylcholine signaling, was indirectly assessed via their ability to inhibit AChE. The screening investigation confirmed that all three polyherbal products exhibited significant, and dose-dependent inhibition of AChE. AChE inhibitory activity was highest (99.7%) with the NHT extract, however this also had the lowest potency (IC_50_ = 324 μg/mL). The other two extracts HCT and PHT displayed AChE inhibition of 61%– 74%, and both with considerable potency (IC_50_ between 0.2 μg/mL–0.7 μg/mL), comparable and indeed slightly lower than the pure drug eserine at 0.9 μg/mL.

The NHT formulation contains *Alchornea cordifolia* (Euphorbiaceae)*, Azadirachta indica* (Meliaceae) and *Pteridium acquilinum* (Dennstaedtiaceae) in the ratio of 1:1:2 with a 4.22% yield upon 50% methanolic extraction. Of these three plants, it is only the aqueous bark extracts of *Azadiracta indica* that have been reported previously to have AChE inhibitory activity (36). Furthermore, *Azadiracta indica* has been reported to be effective in an experimental model of AD by virtue of its cognition enhancement, anti-depressant and anti-anxiety properties (37). Although not reported for *Alchornea cordifolia*, another member of the Eurphorbiaceae family, *Jatropha gossypifolia* L has been reported to exhibit AChE inhibitory activity (38). The role of *Pteridium acquilinum* on memory enhancement is as yet unknown, and may also contribute to the anti-AChE activity we detected for the polyherbal product, but we have yet to investigate this further.

The phytoconstituents of HCT includes *Hippocratea volubilis* (Hippocreateaceae), *Viscum album* (Santalaceae), and *Thymus pulegiodes* (Lamiacene) in the ratio of 2:1:1. A new triterpene caffeoyl ester, lupeol caffeate was obtained from *Hippocratea volubilis* (39) with reported benefits to a broad range of diseases (40). *Viscum album* is reported to exhibit sedative, anti-epileptic and anti-psychotic activity in mice (41), anti-oxidant capacity (4), as well as anti-amyloid β and anti-dementia effects, and therefore may have a therapeutic role in the prevention of the progression of AD (42). *Thymus pulegiodes* is a rich source of natural anti-oxidants and AChE inhibitors, which may be useful in preventing and treating AD and other neurodegenerative disorders (43).

PHT contains *Urtica dioica, Hippocratea volubilis*, and *Thymus vulgaris* in the ratio of 1:2:1. Luteolin, a flavonoid has been isolated from *Thymus vulgaris* and is known to possess anti-amyloid activity (44, 45). *Urtica dioica* has been reported to possess anti-oxidant and anti-microbial activities (46).

An assessment of the anti-oxidant activity via DPPH free radical scavenging and ferric iron reducing capacity demonstrated that NHT was the most active polyherbal agent. Anti-inflammatory (47, 48, 49) and anti-oxidant (50) effects of agents from NHT (*Alchornea cordifolia*) have also been reported. Likewise *Alchornea cordifolia* alone also has a relatively high total phenolic content with useful free radical scavenging activity reported (51).

## Conclusion

The phytocomponents of these polyherbal products possess significant anti-cholinesterase, and anti-oxidant activities (refer to graphical abstract). These inherent pharmacological properties are useful to address multiple elements of the etiopathology of AD, and therefore lay credence to the potential use of ethnomedicine by herbalists for memory enhancing activity. These polyherbal extracts remain a potentially beneficial pharmacotherapy for the management of diseases with cholinergic deficits, such as AD, PD, and myasthenia gravis.

## Figures and Tables

**Table 1:** Name of standardised herbal products, components and yield of methanolic extract

S/No.	Name of Herbal Product	Constituents/ Common name	Family	Composition	Yield (%)
1.	Neocare herbal tea (NHT)	*Alchornea cordifolia* (Christmas bush)	Euphorbiaceae	25%	4.22
		*Azadirachta indica* (Neem leaves)	Meliaceae	25%	
		*Pteridium aquilinum* (Eagle Fern)	Dennstaedtiaceae	50%	
2.	Herbalin complex (HCT)	*Hippocratea volubilis* (Hepocretea pollens)	Hippocrataeceae	50%	5.52
		*Viscum album* (mistletoe)	Santalaceae	25%	
		*Thymus pulegiodes* (Mother thyme)	Lamiaceae	25%	
3.	Phytoblis Herbal Tea (PHT)	*Urtica dioica* (Stinging nettles)	Urticaceae	25%	5.24
		*Hippocretea volubilis* (Hepocretea pollens)	Hippocretaeceae	50%	
		*Thymus vulgaris* (Thyme)	Lamiaceae	25%	
		Aloe vera	Asphodelaceae	25%	

**Table 2:** AChE inhibitory and DPPH radical scavenging activities of polyherbal extracts

S/No.	Polyherbal Methanolic Extracts / pure drugs	Inhibitory concentration (IC_50_)	
		Anticholinesterases activity	Antioxidant activity
1.	NHT	324.0 ± 3.9	0.01 ± 0.023
2.	HCT	0.20 ± 0.7	0.08 ± 0.0032
3.	PHT	0.70 ± 2.9	0.30 ± 0.0020
4.	Eserine	0.90 ± 0.03	-
5.	α-Tocopherol (vitamin E)	-	20.0 ± 0.002

All values are reported as means ± SD (*n* = 3). Polyherbal extract inhibition of AChE was measured using a modified Ellman assay, with percentage inhibition of AChE calculated relative to eserine; polyherbal anti-oxidant activity was assessed via the percentage inhibition (radical scavenging) of DPPH with vitamin E used as a positive control.

**Table 3:** Effect of standardised polyherbal methanolic extracts on HepG2 cells viability

S/No.	Polyherbal Extracts	1 μg/mL	10 μg/mL	100 μg/mL	1000 μg/ mL	IC_50_ (μg/mL)
1.	NHT	111.3±3.5^a^	107.5±9.2	70.1±6.3^b^	43.2±5.22^c^	~745
2.	HCT	105±5	103.5±7.25	91.6±6.4	65.65±9.34^c^	~1400
3.	PHT	104.7±7.5	102.4±3.4	95.4±6.36^a^	71.2±5.3^c^	>1650

HepG2 cells were incubated with polyherbal extracts at the specified concentration for 48 h and the percentage of viable cells evaluated using a MTT assay. Polyherbal extracts were assessed at least in triplicate across a 1 μg/mL–1000 μg/mL concentration range, and an approximate IC_50_ calculated.

All values are reported as means (±SD) (*n* = 3)

a *P* = 0.043,

b *P* = 0.006,

c *P* = 0.0001.
